# Guidelines on lung adenocarcinoma prognosis based on immuno-glycolysis-related genes

**DOI:** 10.1007/s12094-022-03000-9

**Published:** 2022-11-29

**Authors:** Yuting Zhang, Wen Qin, Wenhui Zhang, Yi Qin, You Lang Zhou

**Affiliations:** 1 Department of Thoracic Surgery, Affiliated Hospital of Nantong University, Medical School of Nantong University, Nantong, 226001 Jiangsu China; 2 Nantong Key Laboratory of Translational Medicine in Cardiothoracic Diseases, Research Institution of Translational Medicine in Cardiothoracic Diseases, Affiliated Hospital of Nantong University, Nantong, 226001 Jiangsu China; 3 Research Center of Clinical Medicine, Affiliated Hospital of Nantong University, Nantong, 226001 Jiangsu China; 4 Nursing Department, Affiliated Hospital of Nantong University, Nantong, 226001 Jiangsu China

**Keywords:** Lung adenocarcinoma, Glycolysis, Immune, Prognosis, Bioinformatics

## Abstract

**Objectives:**

This study developed a new model for risk assessment of immuno-glycolysis-related genes for lung adenocarcinoma (LUAD) patients to predict prognosis and immunotherapy efficacy.

**Methods:**

LUAD samples and data obtained from the Cancer Genome Atlas (TCGA) and Gene Expression Omnibus (GEO) databases are used as training and test columns, respectively. Twenty-two (22) immuno-glycolysis-related genes were screened, the patients diagnosed with LUAD were divided into two molecular subtypes by consensus clustering of these genes. The initial prognosis model was developed using the multiple regression analysis method and Receiver Operating characteristic (ROC) analysis was used to verify its predictive potential. Gene set enrichment analysis (GSEA) showed the immune activities and pathways in different risk populations, we calculated immune checkpoints, immune escape, immune phenomena (IPS), and tumor mutation burden (TMB) based on TCGA datasets. Finally, the relationship between the model and drug sensitivity was analyzed.

**Results:**

Fifteen (15) key differentially expressed genes (DEGs) with prognostic value were screened and a new prognostic model was constructed. Four hundred and forty-three (443) samples were grouped into two different risk cohorts based on median model risk values. It was observed that survival rates in high-risk groups were significantly low. ROC curves were used to evaluate the model’s accuracy in determining the survival time and clinical outcome of LUAD patients. Cox analysis of various clinical factors proved that the risk score has great potential as an independent prognostic factor. The results of immunological analysis can reveal the immune infiltration and the activity of related functions in different pathways in the two risk groups, and immunotherapy was more effective in low-risk patients. Most chemotherapeutic agents are more sensitive to low-risk patients, making them more likely to benefit.

**Conclusion:**

A novel prognostic model for LUAD patients was established based on IGRG, which could more accurately predict the prognosis and an effective immunotherapy approach for patients.

**Supplementary Information:**

The online version contains supplementary material available at 10.1007/s12094-022-03000-9.

## Introduction

At present, Lung cancer has become the second most common cancer worldwide, and its incidence and deaths are increasing over the years with a high level of invasiveness. In 2020, Lung cancer accounted for 11.4% of all new cancer cases, 18.0% (1.8 million) of deaths from malignant tumors worldwide [1]. Non-small cell lung cancer (NSCLC) accounts for about 80–85% of primary lung cancers [2], with 70% of patients having progressed to intermediate or advanced/late stages by the time of diagnosis [3]. Lung adenocarcinoma (LUAD) accounts for approximately 40% of all newly diagnosed lung cancer cases and is the most common histologic form of NSCLC [4, 5]. Despite significant advances in non-invasive surgery and immunotherapy in recent decades, 5-year overall survival (OS) rate is still as low as 17.4% [6]. An accurate prognosis of LUAD patients is a prerequisite for more effective treatment, improved survival rates and reduced mortality, which remains a great clinical challenge to the current medical healthcare system.

Cellular metabolism involves the synthesis, maintenance or decomposition of biomolecules, which not only provide material and energy for cell activities but also function as signaling transduction agents or transducers. Abnormal metabolic reprogramming promotes cell growth and division, leading to uncontrolled and sustained malignant proliferation [7]. When metabolic reprogramming occurs, it can cause various diseases, such as glycolysis disorders, which can lead to diabetes and various cancers [8, 9]. Numerous specific metabolic reprogramming occur during precancerous lesions, for example, the oncogenic KRas gene causes metabolic reprogramming, which increases mitochondrial reactive oxygen species (mROS) and promotes acinar ductal metaplasia (ADM), which contributes to the development of pancreatic cancer [10]. Therefore, metabolic reprogramming can be used to predict the occurrence of cancer under certain conditions, and focusing on metabolic markers in tumor metabolic reprogramming has functional and meaningful implications for targeted therapy.

Abnormal glucose metabolism is an important part of tumor metabolic reprogramming. Tumor cells alter metabolic fluxes to maintain their normal survival and progression in the microenvironment. Tumor cells do not metabolize energy through oxidative phosphorylation (OXPHOS), which is significantly different from normal cells. One of the most common and important changes to metabolism is aerobic glycolysis, widely referred to as the “Warburg effect”. Warburg effect represents a shift in glucose utilization by tumor cells from oxidative phosphorylation to glycolysis, characterized by increased glucose uptake and lactate secretion, occurs even under normal oxygen content, and is now considered as one of the hallmarks of tumors [11–13]. Many glycolysis-related enzymes, such as hexokinase 2, phosphofructokinase, and pyruvate kinase, are overexpressed in lung cancer cells compared to normal cells [14–16]. The reprogramming of metabolic pathways facilitates the malignant proliferation of tumor cells and the ability to adapt to the harsh living environment, providing energy and conditions for the proliferation and invasion of cancer cells in LUAD [17].

Tumor microenvironment (TME) is an integrated cellular environment surrounding a tumor cell in which various innate immune cells. When the tumor microenvironment is associated with the function and signal transduction of these immune cells, it can also be refered to as the tumor immune microenvironment (TIME). Dysregulation of the tumor immune microenvironment and alterations of metabolic pathways are two unique markers of tumor cells [18]. The tumor microenvironment, especially the immune microenvironment, is a key factor in evaluating the clinical survival of cancer patients and can effectively reflect the ability of immune response [19, 20]. In TIME, tumorigenesis and evolution are important as crosstalk between immune cells and tumor cells generates an environment that promotes tumor proliferation and metastasis. For example, PD-1 on the surface of T cells interacts with PD-L1 on the surface of tumor cells, inhibiting T cell immune function and protecting tumor cells from immune attack, resulting in an immune evasion [21]. Thus, the state of the immune microenvironment can determine tumor cell progression and anti-tumor immune response.

In this study, a new prognostic feature based on glycolysis-related genes (GRGs) and immune-related genes (IRGs) was developed and characterized by multiple statistical methods showing their reliability. It improves the ability to accurately determine the prognosis of LUAD and provides assistance for the rescheduling of clinical management strategies.

## Materials and methods

### Data acquisition and collection

Data collected for lung adenocarcinoma mRNA expression and clinical data were obtained from TCGA (https://www.portal.gdc.cancer.gov/)—LUAD dataset, and microarrays obtained at GEO (https://www.ncbi.nlm.nih.gov/geo/) were used for validation. The following analysis was performed using the expression profile of 594 LUAD samples (535 tumors and 59 normal). Clinical information of LUAD patients was downloaded from TCGA-LUAD dataset, including survival time and status, clinical grade, gender, age, TMN classification. The information from 488 LUAD patients was later used for model development and accuracy validation, excluding patients with 0 survival time and incomplete information. The expression matrix file (GSE68465) from the GEO database was then used for external validation. Three hundred and two (302) GRGs were obtained by accessing GSEA (http://www.gsea-msigdb.org/gsea/index.jsp), with 2483 IRGS available in ImmPort (https://www.immport.org/). These data sources are publicly accessible, so the study has no ethical or conflict of interest and does not require review approval from a local council.

### Acquisition of intersecting genes

The obtained three hundred and two (302) glycolysis-related genes and 2483 immune-related genes were used to draw a Venn diagram using an online tool (http://www.bioinformatics.psb.ugent.be/webtools/Venn/). Twenty-two (22) overlapping genes were identified from the two sets of data, overlapping genes were identified as candidate genes for subsequent analysis.

### Screening for immune- and glycolysis-related DEGs

Differentially expressed genes (DEGs) were calculated using the “Limma” R package (version 4.1.2) to identify which immuno-glycolytic related genes were differentially expressed in normal and tumor tissues. Genes with *P* < 0.05 were identified as DEGs by the Wilcoxon rank sum test. The “pheatmap” package in R language was used to visualize the DEGs and draw the heatmap. The protein–protein interactions (PPI) of DEGs were calculated using the online public database STRING database (version11.5, https://www.string-db.org), setting the confidence score to ≥ 0.4 and removing free nodes. The PPI network was drawn next to elucidate the protein–protein interactions. The correlation coefficients between DEGs were calculated after removing samples from normal tissues via the “igraph” package in R language, and a co-expression network graph was created, which finally showed the interrelationships between 11 DEGs.

### Consensus clustering

The “ConsensusClusterPlus” tool in R was used to implement an unsupervised clustering method that divides the LUAD samples in the TCGA dataset into two groups based on 22 prognostic candidate genes. The ideal number of clusters between *k* = 2 and 9 was then evaluated and 1000 replications/repetitions were performed to determine the most reliable classification. Based on the “survival” and “survminer” packages in R language, survival differences between the different clusters were analyzed, and *P* < 0.05 was considered as the difference in patient survival between the two clusters. This was then visualized using the “ggsurvplot” package to plot Kaplan–Meier (K–M) survival curves, DEGs between different clusters were identified using the “ggplot2” package in R language. Twenty-two (22) candidate genes were simultaneously observed for DEGs between different clusters (FDR < 0.05, |logFC|> 1) and heat maps were created for 7 DEGs screened by pheatmap to visualize their differential expression and clinical traits between clusters.

### Risk model construction and validation

For genotyping differential genes, univariate Cox regression analysis was performed using the “survival” package to screen out genes associated with OS (*P* < 0.05) and identified as prognosis-related genes. Then “glmnet” was applied to process the above-mentioned genes to identify key genes and build a prognostic model, thereby selecting the optimal number of genes and candidate genes by the obtained least absolute shrinkage and selection operator (LASSO) results. The formula of risk score obtained according to LASSO regression results was as follows: $\mathrm{risk} \mathrm{score}=\sum_{i=1}^{n}\left({\mathrm{Coef}}_{\mathrm{i}}\times {\mathrm{Exp}}_{\mathrm{i}}\right)$

Where *n* is the number of prognosis-related genes in the model, Coef_i_ the related gene coefficient, and Exp_i_ represents gene expression. All patients included in the analysis were grouped into high or low risk according to the cut-off point of the best risk score. Kaplan–Meier(K–M) curves were plotted using the “survminer” R package to detect and demonstrate differences in survival rates. Receiver operating characteristic (ROC) curves were also plotted at 1, 3, and 5 years to validate the accuracy of the prognostic model developed. A dot plot was created with “pheatmap” in R to determine the association between risk score and survival status. Principal Component Analysis (PCA) and t-distributed Stochastic Neighbor Embedding (t-SNE) analysis were performed using “ggplot2” and “Rtsne” to explore whether the model could accurately distinguish between different risk groups and visualize the results. This was followed by univariate and multivariate analyses combining risk scores with clinical characteristics to explore the correlation between this index and patient OS, and those with significant correlation were identified as independent prognostic factors (*P* < 0.05).

### Pathway enrichment and immune function analysis of differential genes

Gene ontology (GO) annotation and immune infiltration were evaluated according to DEGs (|logFC|≥ 1 and FDR < 0.05) in both risk groups using the “clusterProfiler” and “gsva” R software packages. Filtered with *P* value < 0.05 and *q* value < 0.05 as thresholds to identify significant enrichment pathways. The Single sample gene set enrichment analysis (ssGSEA) was assessed for potential immunological function and active pathways for relevant biological values.

### Immune response and tumor mutation burden analysis

Potential immune checkpoints were extracted from previous literature reviews, and the “ggpubr” R package evaluated and compared the expression levels of 22 immune checkpoint genes in the high and low-risk groups. The correlations between immune-related genes and risk scores were then assessed using spearman correlation analysis, and correlation analysis was performed using the R package “limma”. The TME of both groups was also analyzed. The Cancer Immunome Atlas (TCIA, https://www.tcia.at) provided an Immunophenoscore (IPS) for LUAD patients. In combination with the Tumor Immune Dysfunction and Exclusion (TIDE) algorithm, the predictive efficiency of the model for immunotherapy response was analyzed in the high-risk and low-risk groups. Immune evasion and correlation analyses were performed using the “ggpubr” and “corpplot” software packages, respectively. The differences in methylation expression between the two risk groups were further compared and the immune score, stromal score, estimated score and tumor purity were analyzed for both groups. Based on the downloaded nucleotide variant data from LUAD, the mutational load of the samples was calculated using perl software (version 5.32.1), thus comparing the differences between the two risk groups and evaluating the correlation between mutational load and risk. Next, the mutational burden and the risk value were combined and evaluated for survival analysis using “survminer” R.

### Sensitivity analysis of chemotherapy

Treatment response to known common chemotherapeutics was assessed using the “pRRophetic” package. The half-maximal inhibitory concentrations (IC50) were calculated from the TCGA-LUAD dataset to investigate the difference in sensitivity to commonly used chemotherapeutic drugs between the high and low expression groups, and thus estimate the relationship between the model and drug response.

### Statistical analysis

All statistical and graphing work was done by R software (4.1.2). Perl was used for all data processing and collation of the data matrix. The K–M method and Log-rank test were used to analyze survival curves and differences. Univariate and multivariate Cox analyses were performed to determine whether the prognostic model could be used as an independent prognostic factors. Differences between the two groups were compared using the Wilcoxon rank sum test, Spearman’s correlation analysis method was used to assess the correlation and all heatmaps were generated by the pheatmap parameter in R software. Statistical tests were two-sided, and *P* < 0.05 was considered a criterion to distinguish differences.

## Results

### Analysis of genes related to immunity and glycolysis

**Fig. 1:**
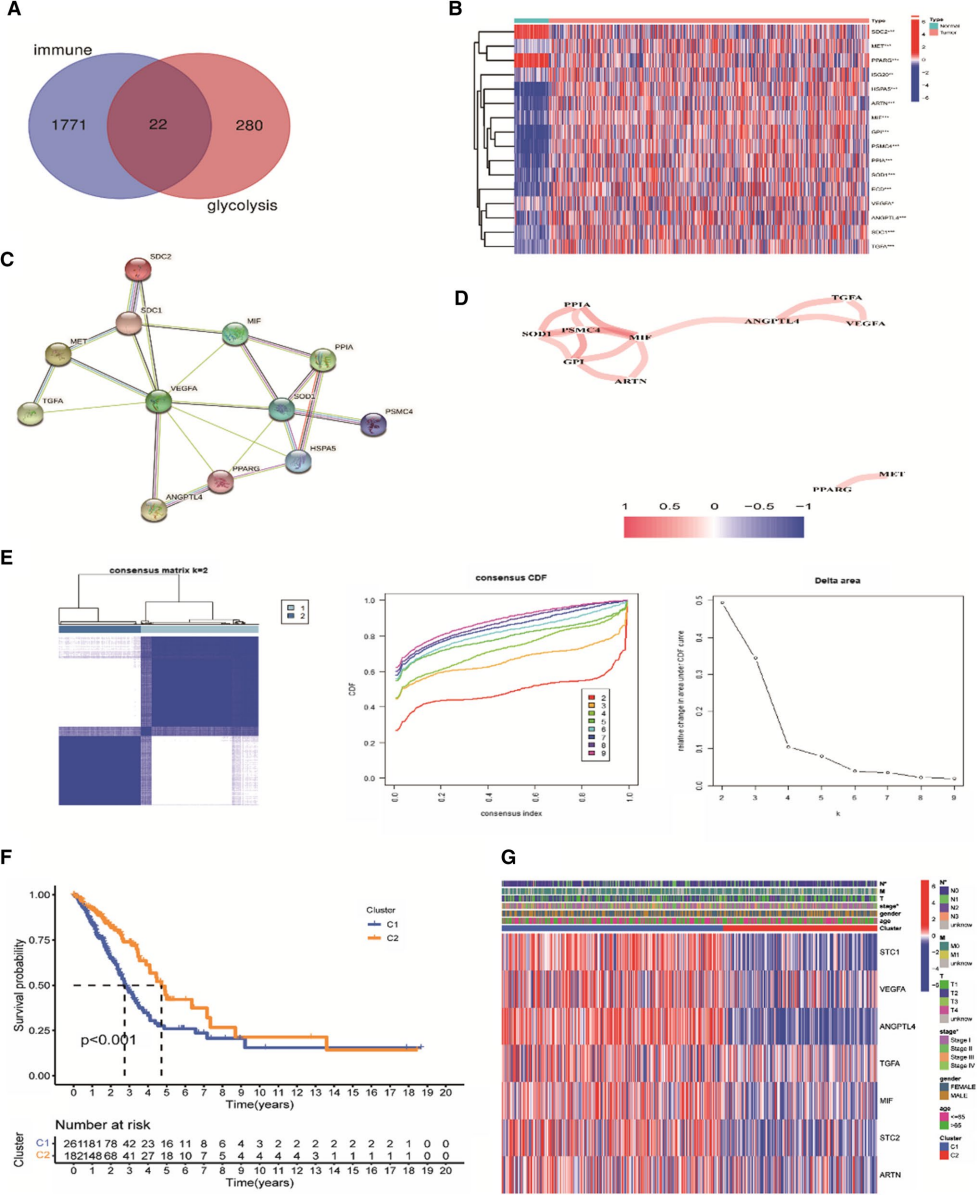
Analysis of genes related to immunity and glucose metabolism and classification of clusters. **A** A Venn diagram shows the intersection of immune-glycolysis-related genes. **B** Heatmap of 16 differentially expressed genes in normal samples and LUAD. *P < 0.05; **P < 0.01; ***P < 0.001. **C** The network is made up of 12 interconnected, differentiated genes. **D** The co-expression network consisted of 11 positively correlated genes (red: positively correlated; Blue: negative correlation). **E** Consensus clustering matrix, CDF curves and the relative changes of different clusters under the CDF curve in TCGA cohort. **F** Kaplan–meier curves of OS of two groups of LUAD patients and the number of surviving patients in cluster1 and cluster2 at different time periods. **G** Heatmap and clinicopathological characteristics of differential genes in two clusters (cluster1 and cluster2) (*P < 0.05)

A Venn diagram was drawn for 302 GRGs and 2483 IRGs, 22 candidate genes (MET, GPI, SDC2, PPARG, PSMC4, PPIA, VEGFA, ANGPTL4, SOD1, SDC1, HSPA5, ISG20, TGFA, MIF, ECD, ARTN) were obtained as shown in Fig. 1A. Twenty-two (22) IGRGs were differentially analyzed between tumor and normal tissues, among them, 16 genes showed differential expression, with SDC2 and PPARG significantly down-regulated in tumor samples. MET, GPI, ANGPTL4, HSPA5, TGFA, MIF, and ARTN were significantly up-regulated in tumor samples (Fig. 1B). A protein–protein interaction (PPI) network analysis was established for these 16 differential genes using the STRING database to identify their interactions. Four free nodes (ARTN, IAG20, ECD and GPI) were removed to obtain the protein interaction between the remaining 12 genes (Fig. 1C),co-expression networks of 16 DEGs were subsequently constructed by weighted gene co-expression network analysis, and the results showed that co-expression relationships existed between 11 genes, and all were positively correlated as shown in Fig. 1D.

### Consensus clustering was used to identify two molecular subtypes

The clinical samples in the TCGA database were divided into different clusters (*k* = 2–9) based on the expression of 22 genes, and the consensus matrix, the consensus CDF curve, and the relative change in area under the curve (Fig. 1E) showed that *k* = 2 was the optimal partition. Four hundred and forty-three (443) clinical data of lung adenocarcinoma were rationally assigned to two different subtypes named Cluster1 (C1, *n* = 261) and Cluster2 (C2, *n* = 182). K–M survival analysis subsequently showed that Cluster1 had an inferior survival rate compared to Cluster2 (*P* < 0.001) (Fig. 1F). This also implied that the median clinical survival was higher in Cluster2 patients. Moreover, 7 DEGs (STC1, VEGFA, ANGPTL4, TGFA, MIF, STC2 and ARTN) were obtained by differential genetic analysis of 22 IRGs in the two genotypes. The clinicopathological characteristics of these seven differential genes between the two subtypes were then investigated. Heatmap results showed that all 7 genes were down-regulated in Cluster2, with different subtypes distributed differently in the N stage (*P < 0.05) and the clinical stage (*P < 0.05) (Fig. 1G).

### Prognostic model construction and validation

**Fig. 2:**
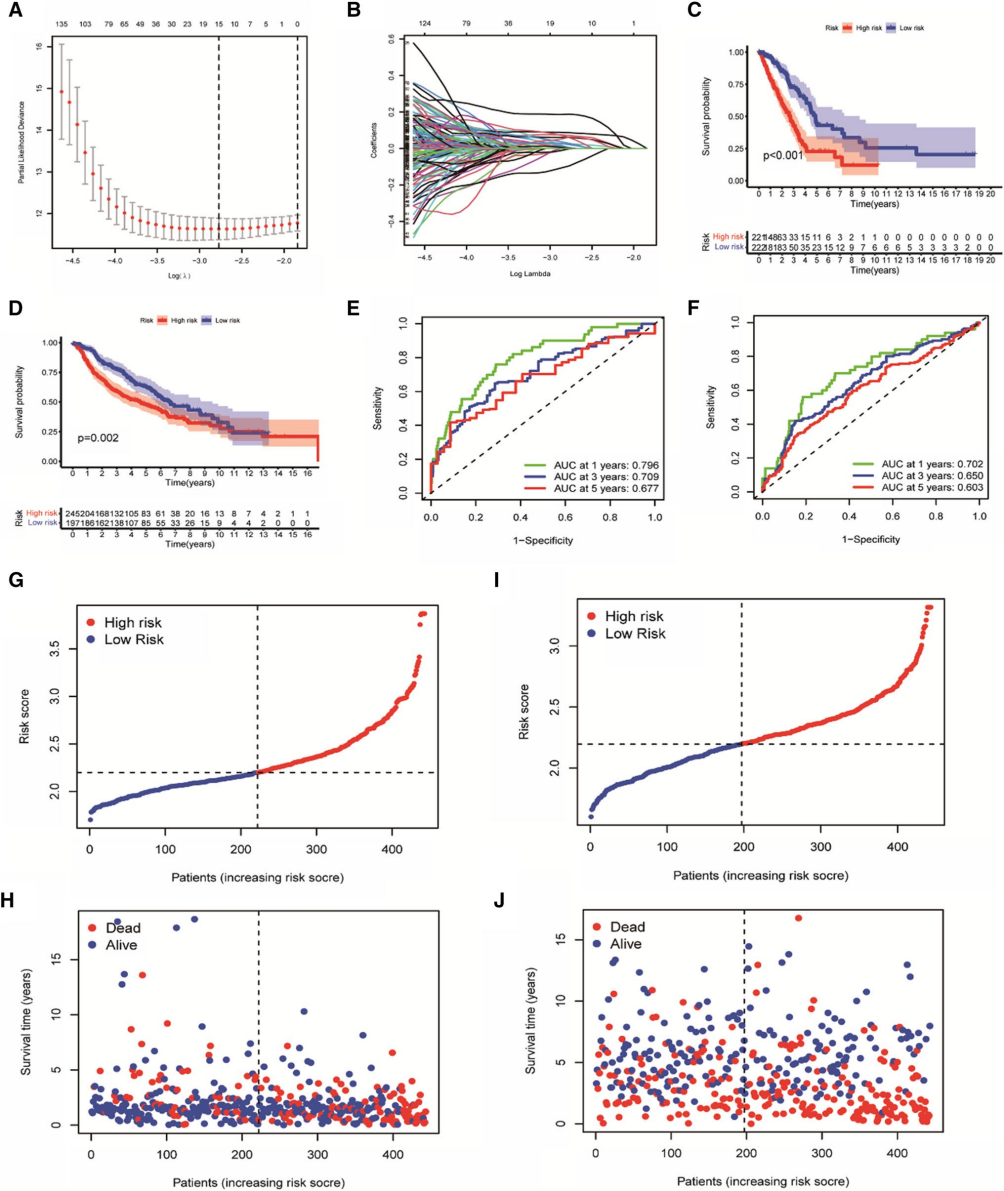
Establishment and validation of IGRGs prognosis model based on the training set. **A**–**B** Regression coefficients and partial likelihood deviations of 393 prognostic DEGs. **C**–**D** K–M analysis of two risk subgroups of the training group and validation group and the number of patients in the two groups who survived in different time periods. **E**–**F** The model predicts AUC for patient survival. **G**–**H** Scatter plot of the score distribution, survival time and status of patients in the training set. **I**–**J** Risk score distribution, survival time, and status in the validation set

Differential expression analysis was first performed on Cluster1 and Cluster2, and a total of 1567 DEGs were identified. These DEGs were used for batch correction and expression extraction in TCGA and GEO databases. The TCGA and GEO expression data were combined with the survival data after excluding normal samples. The TCGA cohort was used as the training group and GES68465 from the GEO database was used as the test group. Three hundred and ninety-three genes (393) genes were then selected as prognostic genes from the combined TCGA survival and expression data (Supplementary Table 1). To avoid the risk of over-fitting and subsequent bias, the Cox regression model of the lasso method was optimized for the above 393 genes, of which 15 genes were identified as optimal variables (Fig. 2A, B). Finally, these 15 genes were used to construct the prognosis model, and the following equation was obtained: Risk score = 0.0717 × FLNC + 0.0025 × FBN2 + 0.0054 × CCL20 + 0.1881 × NTSR1 + 0.0265 × KRT6A + 0.0869 × DKK1 + 0.0004 × KYNU + 0.0605 × TENM3 + 0.0011 × ANGPTL4 + (− 0.0952) × STAP1 + 0.0606 × HMMR + 0.0282 × IGFBP1 + (− 0.0013) × C11orf16 + 0.0623 × LDHA + 0.0209 × PLEK2. LUAD patients in the training group were divided into high (*n* = 221) low (*n* = 222) risk groups based on optimal cutoff values.

K–M analysis showed a significant reduction in survival rate for all high-risk patients in the training and test groups. There was a significant difference in overall survival between the two risk cohorts in the training group (*P* < 0.001), while the low-risk cohort had significantly better survival outcome. This conclusion was also supported by the test group (*P* = 0.002) (Fig. 2C, D). The effectiveness of the risk score in predicting OS was assessed by ROC analysis, the area under the curve (AUC) in the training group were 0.796, 0.709, and 0.677, in the test group, they were 0.702, 0.650, and 0.603, at 1, 3 and 5 years, respectively. These two sets of results showed that the model was highly specific and had the good predictive ability (Fig. 2E, F). The accuracy was then validated, and a survival distribution was plotted for the training and test groups to explore the relationship between risk values and survival prognosis (Fig. 2G–J). These results showed that as risk increased, mortality increased and survival decreased.

### Analysis of independent prognostic factors in training and test groups

**Fig. 3:**
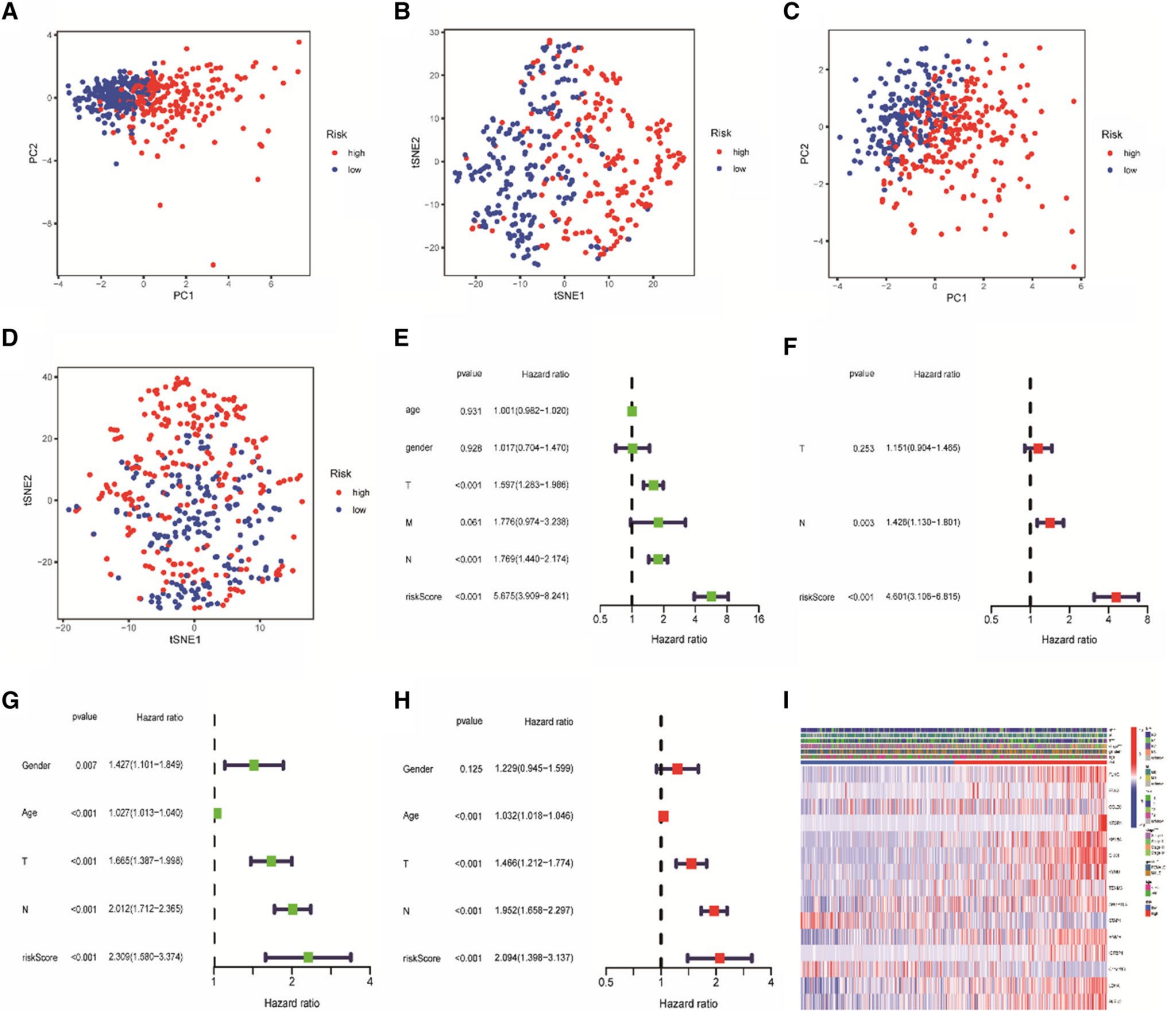
Independent prognostic value of gene characteristics in training and test cohorts. **A**–**D** PCA and t-SNE analysis of the model. **C**–**D** PCA and T-SNE analyses based on prognostic genes in the validation cohort. **E**–**F** Univariate and multivariate analyses of clinical characteristics associated with survival in the training cohort. **G**–**H** Univariate and multivariate analysis of OS-related factors in the validation cohort. **I** Risk heatmaps of 15 prognostic genes. *P < 0.05; **P < 0.01; ***P < 0.001

Each patient's clinicopathological features were analyzed and validated. The model had reliable clustering ability in both groups as revealed by principal component analysis (PCA) and t-distributed stochastic neighbor embedding (t-SNE) (Fig. 3A–D). Univariate analysis revealed clinical parameters, such as T (*P* < 0.001) and N (*P* < 0.001), were closely related to the OS of the training group, the risk score (*P* < 0.001) was an independent prognostic factor for LUAD, with a hazard ratio (HR) of 5.675 (Fig. 3E). Multivariate Cox regression results obtained revealed that the risk score (*P* < 0.001, HR = 4.601) had the ability to independently predict patients’ OS (Fig. 3F). Also, similar results were obtained from the GSE68465 test data, where the risk score (*P* < 0.001, HR = 2.094) was also proven to be an independent predictor of poor outcome. (Fig. 3G, H). The expression changes of 15 key prognostic genes in different parameters were then compared in the training group, the heatmap of clinical characteristics showed significant differences in gender (*P* < 0.05), grade (*P* < 0.001), N (*P* < 0.001), and T (*P* < 0.001) between the two groups. Thirteen (13) genes (FLNC, FBN2, CCL20, NTSR1, KRT6A, DKK1, KYNU, TENM3, ANGPTL4, HMMR, IGFBP1, LDHA and PLEK2) were high-risk genes, and two genes (STAP1 and C11orf16) were low-risk genes as shown in (Fig. 3I).

### Functional analysis and immune cell infiltration

**Fig. 4:**
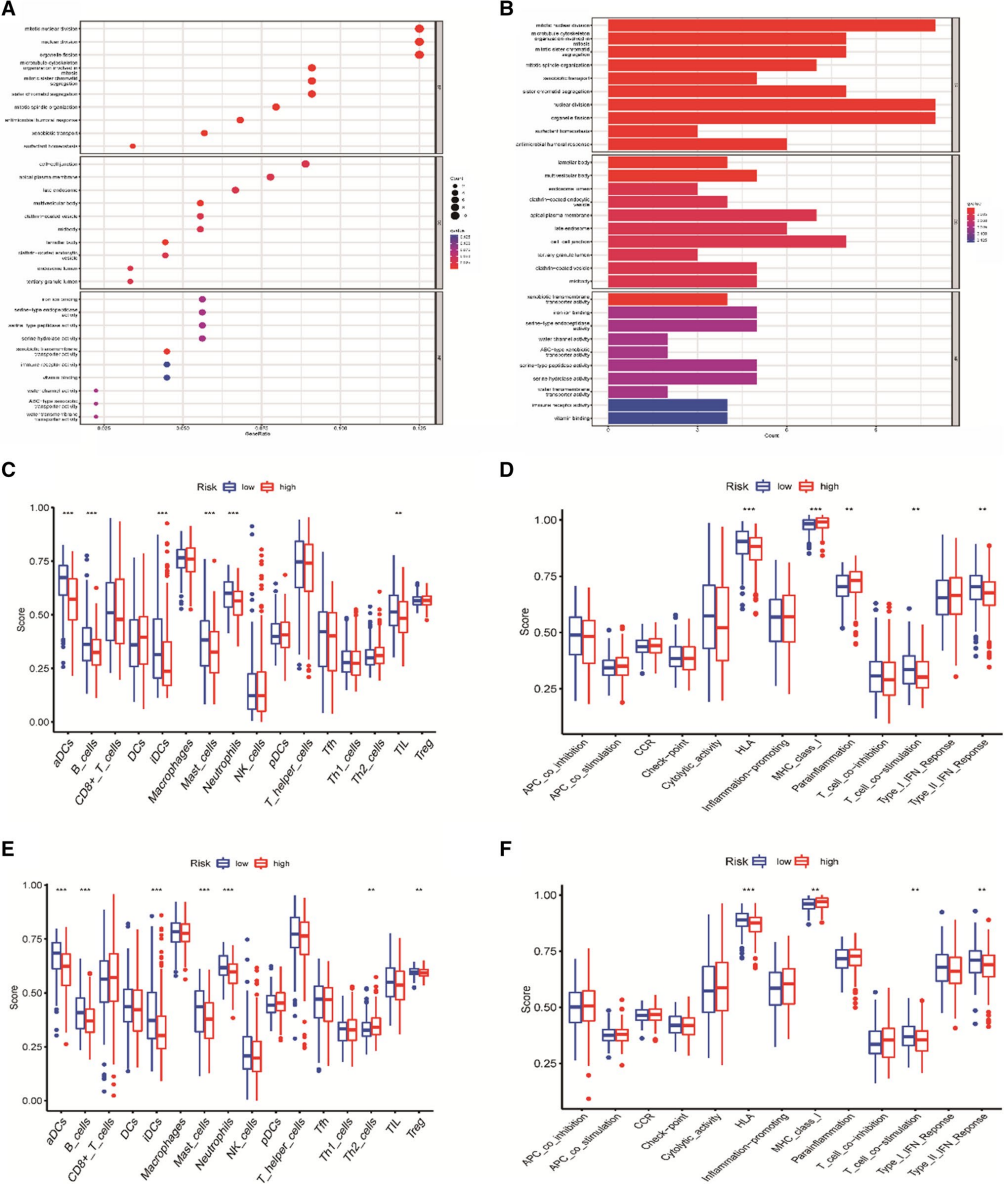
Immune cell infiltration and functional analysis. **A**–**B** GO enrichment analysis based on TCGA queue. **C**–**D** Box plot of immune infiltration and function scores in the TCGA cohort. **E**–**F** Differences in immune infiltration and functional scores between the two risk groups in the validation set

GO analysis was performed on related genes in the training group to further understand the biological functions of risk differential genes in TCGA-LUAD samples. Using “limma” R, DEGs meeting the filtering conditions of FDR < 0.05 and |logFC|> 1 were extracted. Ninety-one (91) DEGs were screened out from 443 genes in the training risk group, among which 44 genes were considered to be pro-oncogenes and 47 anti-oncogenes (Supplementary Table 2). The GO enrichment results showed that DEGs were mainly involved in the biological process of mitosis, nuclear division and organelle fission, and in cytological components mainly associated with cell–cell junctions (Fig. 4A, B).

The relationship between immune environment and risk score was further discussed from an immunological perspective, single-sample gene set enrichment analysis (ssGSEA) was used to assess differences in the functional activity of immune cells and pathways. In the TCGA cohort, immature dendritic cells (iDCs), B-cells, activated dendritic cells (aDCs), neutrophils, mast_cells, tumor-infiltrating lymphocytes (TIL), human leukocyte antigen (HLA), T cell co-inhibition, Type_II interferons(IFN) response were infiltrated at high levels in a low-risk cohort, but MHC_class_I and Parainflammation decreased significantly (Fig. 4C, D). In the GEO cohort, iDCs, ADCs, mast_cells, B-cells, neutrophils, regulatory T cells (Treg), HLA, Type_II_IFN response, T cell co-stimulation were higher in low-risk patients, while Th2_cells and MHC class I scores decreased (Fig. 4E, F). The results were similar to those of TCGA.

### Tumor immune response and mutation burden analysis

**Fig. 5:**
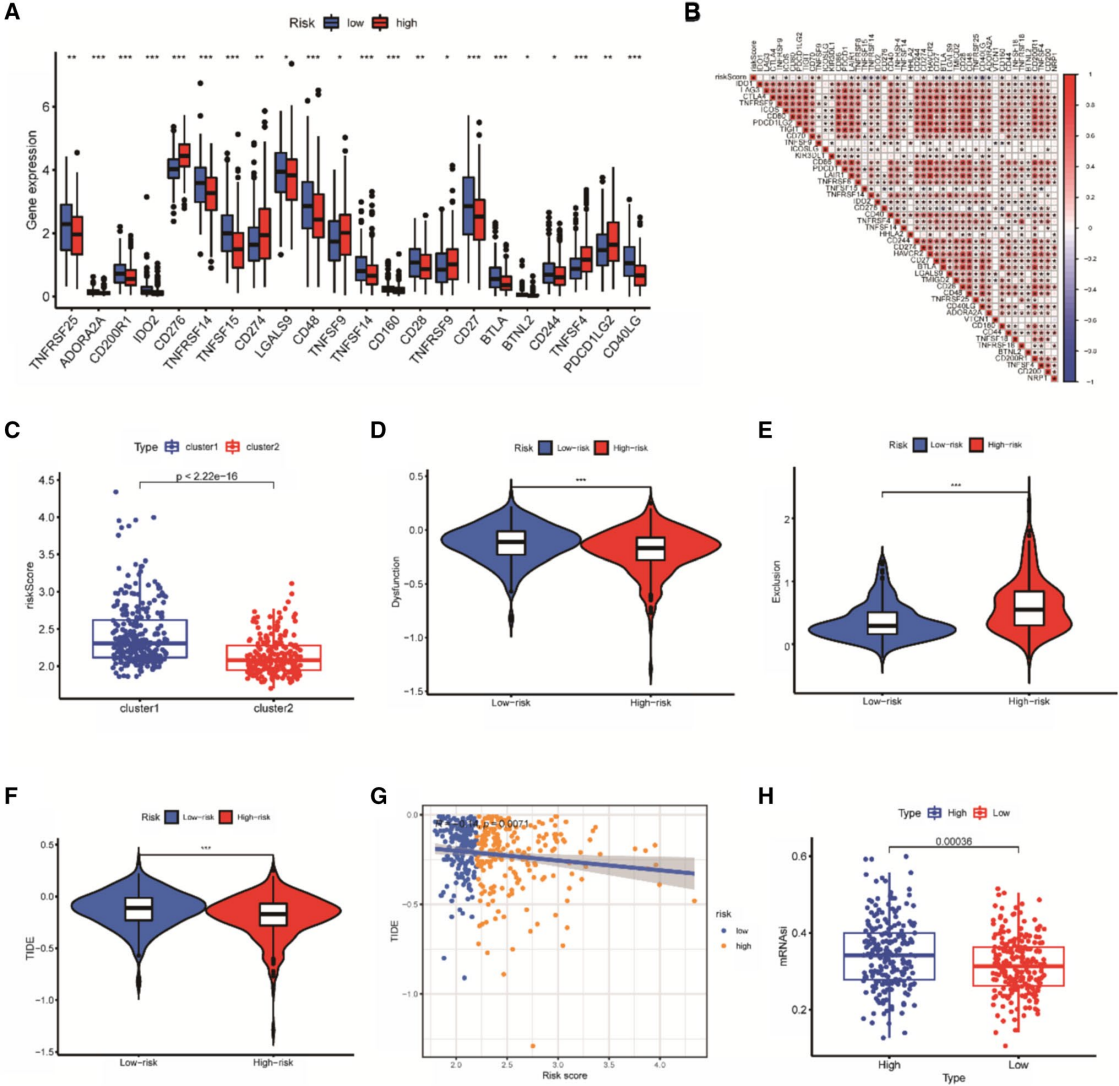
Comprehensive assessment of immune characteristics in TCGA-LUAD dataset. **A** The expression levels of 22 differentially expressed checkpoint genes in two groups of patients. *P < 0.05; **P < 0.01; ***P < 0.001. **B** Association of risk scores with immune checkpoint related genes. **C** Comparison of risk scores between clusters. **D**–**F** The expression levels of tumor immune dysfunction, rejection and microsatellite instability in the two groups were evaluated. **G** Correlation analysis between TIDE fraction and model. **H** Differential expression of mRNAsi

To better understand the status of the immune microenvironment associated with the newly developed risk model, ESTAMATE was performed to calculate Immune scores, stromal scores, ESTIMATE scores, and tumor purity for each risk group, all scores of the two groups compared had no significant differences (Supplementary Fig. S1A–D). In clinical practice, immune checkpoint inhibition is an important approach to cancer treatment. It is an inhibitory molecule that modulates immune activation and kills tumor cells through co-inhibition or co-stimulation. Forty-seven(47) immune checkpoint-associated genes were selected to investigate the relationship between risk models and these genes, the expression of 22 immune checkpoints between different risk groups was studied and observed that most of these had higher levels in the low-score groups (Fig. 5A), correlation analysis showed that there were significant positive correlations among many genes, while the negative correlation between CD40LG and risk scores was most pronounced (Fig. 5B). The risk scores of two different subgroups Cluster1 and Cluster2 were compared, and Cluster1 patients had significantly higher scores than Cluster2 patients (*P* < 2.22E−16) (Fig. 5C).

TIDE tool was used to predict the response of the model to immunotherapy, tumor immune dysfunction was significantly higher in the low-score group (Fig. 5D), whereas oncological rejection was significantly lower in the high-risk group (Fig. 5E). However, there was no significant difference in tumor microsatellite instability (MSI) between the two groups (Supplementary Fig. S2A). Moreover, the TIDE score was significantly lower in the high-risk group (Fig. 5F), and there was a significant inverse correlation between TIDE and risk score (Fig. 5G), suggesting patients in the high-risk group would be benefited more from immunotherapy than patients in the low-risk group. There was an unexpected correlation between mRNA expression based-stemness index (mRNAsi), immune cell infiltration and immune checkpoints. Figure 5H shows the high risk group had stronger stem cell characteristics.

**Fig. 6:**
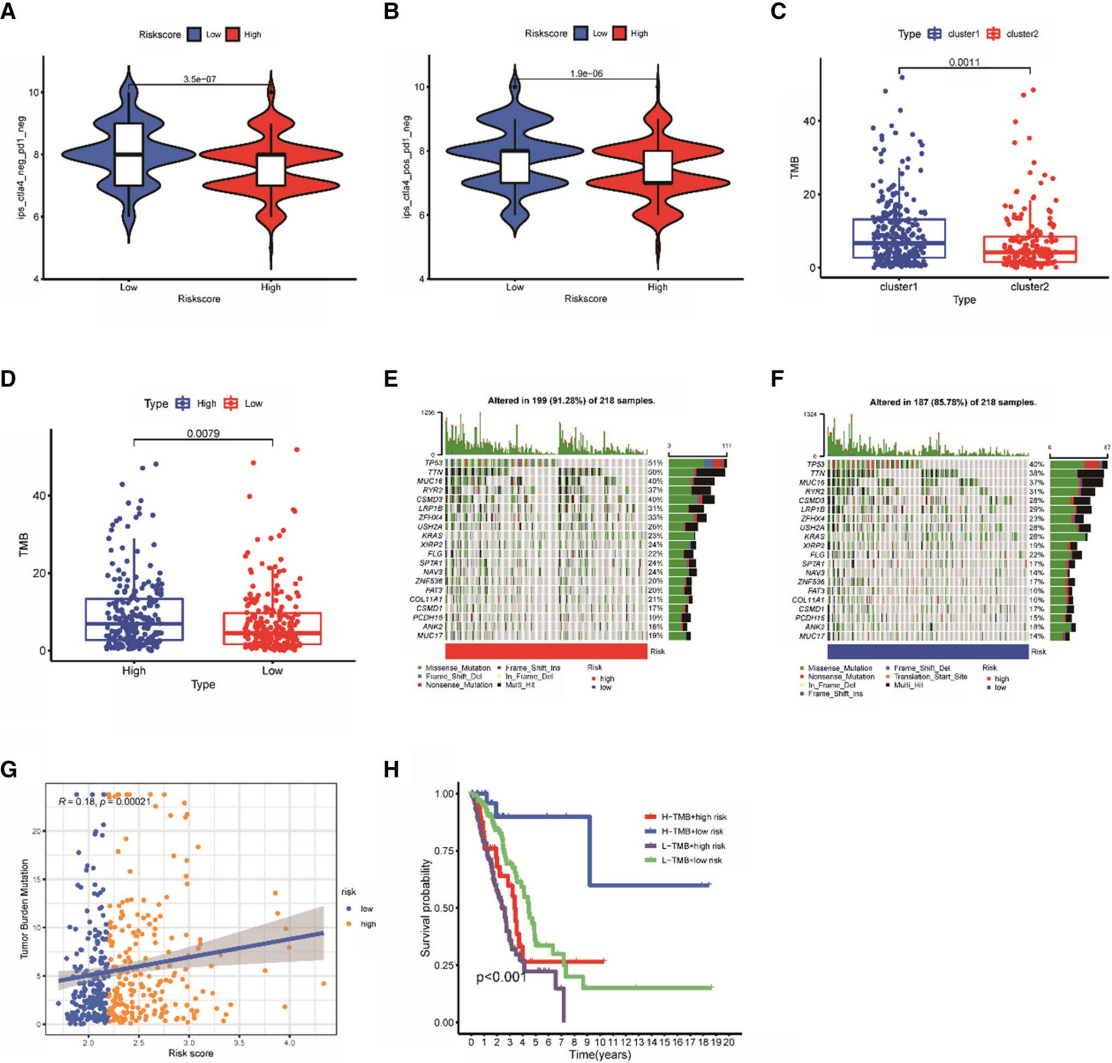
Immunotherapy and evaluation of tumor mutational burden. **A**–**B** Correlation between IPS of two subtypes and risk characteristics. **C** Relationship between the two clusters and TMB score. **D** Boxplot of TMB expression of the training cohort. **E**–**F** Comparison of mutations in the top 20 common genes. **G** Relationship between TMB score and risk score in the training cohort. **H** Survival analyses for patients stratified by both TMB and Riskscore

The immunogenicity of the models was subsequently analyzed using IPS, with higher scores for ips_ctla4_neg_pd1_neg and ips_ctla4_pos_pd1_neg in the low-risk group (Fig. 6A, B), which indicated that low-risk patients had a better response to immunotherapy. However, when PD-1 blockade or its combination with CTLA4 blockade was used, there was no significant difference between the two groups (Supplementary Fig. S2B, C). The TMB expression was then investigated in both clusters and both risk groups, TMB was significantly different between Cluster1 and Cluster2, with Cluster1 having a significantly higher TMB score than Cluster2 (Fig. 6C). The high mutational burden favored the group with the higher score, which was positively and significantly associated with TMB (Fig. 6D). The mutation data in the Training group were used to evaluate the status of TMB in both groups. The top five mutated genes were TP53, TTN, MUC16, RYR2 and CSMD3, and the mutation rate in the high-risk group (91.28%) was higher than that in the low-risk group (85.78%) (Fig. 6E, F). Subsequently, it was observed that higher risk score reflected higher TMB (Fig. 6G). In addition, patients with high tumor mutation burden and low risk had the highest 5-year survival rates (Fig. 6H), while there was no significant difference in 5-year survival between patients with high and low mutations (Supplementary Fig. S2D).

### Analysis of drug susceptibility in two risk groups

**Fig. 7:**
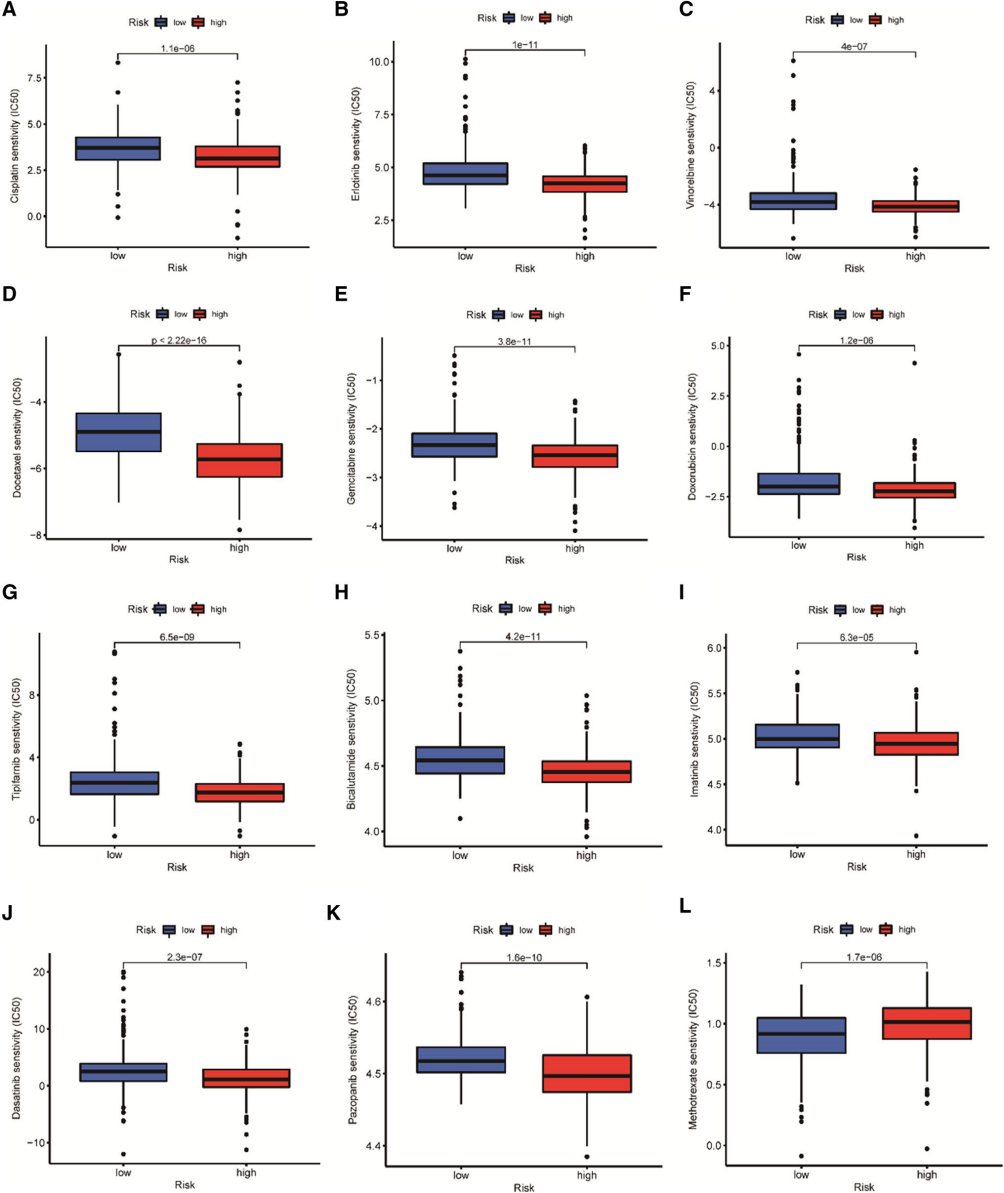
Comparison of chemotherapy response in TCGA-LUAD. **A**–**E** Sensitivity analysis of common chemotherapeutic agents in two risk groups. **F**–**L** Sensitivity analysis of other commonly used chemotherapy drugs in cancer

To further enhance the clinical effect of the risk model, its ability to predict drug sensitivity was investigated. Five (05) common chemotherapeutic drugs (Cisplatin, Erlotinib, Vinorelbine, Docetaxel, Gemcitabine) and seven (07) other cancer chemotherapeutic agents (Doxorubicin, Tipifarnib, Bicalutamide, Imatinib, Dasatinib, Pazopanib, Methotrexate) sensitivities were studied in both groups. The results showed that the low-risk group was more sensitive to cancer chemotherapeutic agents except Methotrexate (Fig. 7A–K), implying that low-risk patients were more sensitive to chemotherapy. As a result, low-risk patients are more likely to benefit from these chemotherapeutic drugs. However, high-risk patients were more sensitive to Methotrexate (Fig. 7L) which had better therapeutic effects.

## Discussion

Cancerous cells show a significant increase in metabolic demands compared to normal cells. Cancerous cells produce more glucose and lactate through a transition pattern from oxidative phosphorylation to glycolysis that promotes proliferation, survival, and metastasis [22]. Glycolysis and the production of lactic acid have been paid more and more attention to tumor immune regulation. Lactic acid has been reported to benefit tumor metastasis, promote angiogenesis, and, more importantly, produce immunosuppression, all of which are associated with poor clinical outcomes [23]. The large amount of lactic acid produced by glycolysis leads to an acidic tumor microenvironment, which facilitates immune evasion [24, 25]. Studies have shown that high lactate concentration in the tumor environment not only inhibits the function of T cells but also inhibits NK and T cells activation, thus realizing tumor cell immune evasion [26, 27]. The lactic acid secreted by the tumor can also impair the cytolysis capacity of CD8 + effector T cells, but Treg cells also need to ingestion lactic acid to maintain their high inhibitory functions [28, 29]. Therefore, glycolysis and immune status may be potential biomarkers of cancer growth, invasiveness and metastasis, and identification of IGRG function may have predictive value for the survival and prognosis of LUAD patients.

Recent studies have shown that cancer markers such as glycolysis and immunity significantly influence the survival prognosis of LUAD. The changes of immune cells affect the occurrence, proliferation and metastasis of tumors [30–32]. A variety of Immunotargeted drugs have also been developed for extensive clinical treatment of cancer [33]. Tumor immunotherapy is an important means, and a series of immune genes with great clinical potential have been discovered (PD-1, CTLA-4) [34, 35], longer OS for some patients. In recent years, the ability of glycolysis to mediate the immune microenvironment has become a focus of attention. In addition, activated T cells are mainly metabolized through the glycolytic pathway, making them play a stronger eradication role. The results show that the glycolysis of immune and tumor cells is not the same and there are differences between them [36]. Although there is an obvious link between glycolysis and immunity, the relationship has rarely been studied in depth.

With advances in bioinformatics and genome sequencing, many models have emerged to assess the potential prognosis of LUAD patients, but most of these analyses are based on genomes or transcriptomes rather than on biological processes. There are increasing evidences that previous clinicopathological factors can no longer meet the need for accurate prediction, additional factors should be considered to synthesize the information. Glycolysis and immune microenvironment are two important biological tumor markers and have great potential in predicting the clinical prognosis of LUAD patients [37, 38]. GRGs and IRGs were included in this study, and IGRG model was constructed through expression data obtained from public databases. IGRG model showed better predictive ability in different subgroups of datasets, and could effectively evaluate the clinical outcome of LUAD patients. Thus, the accuracy of LUAD prognosis indicated by IGRG indicates great potential for clinical application.

In this study, a new prognostic model of IGRG was developed using 15 genes from the TCGA database, among them, CCL20 and DKK1 are IRGs, HMMR and LDHA are GRGs, and ANGPTL4 is both immune and glycolysis-related genes. CCL20 chemokine is a powerful immunomodulatory molecule, commonly present in various mucosal tissues of human body, including liver, lymph node, lung, colon [39, 40], and participates in the regulation of structure and immune homeostasis. CCL20 is a key influencing factor in inflammation and immune response, and CCR6 is the only known chemokine receptor. Some tumors have high expression of CCL20 and its receptor, which proves that CCL20 signal transduction is related to the growth and metastasis of cancerous cells [41, 42]. CCL20 is also involved in controlling the immune response, and has been found to be overexpressed in inflammatory bowel disease (IBD), psoriasis and rheumatoid arthritis, leading to the occurrence of autoimmune diseases (AIDs) [43–46]. CCL20/CCR6 was shown to promote the growth of colorectal cancer through ERK phosphorylation in some studies [47]. A major role of CCL20 in cancer is its involvement in cancer metastasis. One study showed that IL-1β induced signaling pathway can directly stimulate the production of CCL20 in lung cancer cells, and activate MAPK and PI3K signaling pathways through its autocrine, which has a positive effect on the progression and invasion of cancer cells in lung tissue [48]. Another study showed that compared to normal tissues or cells, CCR6 was overexpressed in laryngeal cancer tissues or cell lines. P38 was significantly activated through the CCL20/CCR6 axis, and then p38 played a signal transduction function to modify the miRNA spectrum, thereby creating conditions for the metastasis of tumor cells [49]. Another important role of CCL20 is to determine resistance to treatment. For example, upregulations of CCL20 are associated with gefitinib resistance, and CCL20 can be used as a biomarker to predict gefitinib resistance [50]. Therefore, CCL20 can be used as an effective biomarker for the clinical monitoring of LUAD patients.

DKK1 is a secretory glycoprotein with stronger inhibitory effects on the Wnt/β-catenin signaling pathway and is also an endogenous Wnt signaling antagonist. As a member of a typical carcinogenic signaling pathway, DKK1 plays an important anticancer role in human cancers [51, 52]. Evidence has shown that DKK1 is not only involved in osteogenesis but also plays a central role in promoting tumor bone metastasis [53, 54]. In the tumor tissue of thyroid papillary carcinoma (PTC), abnormal nuclear localization of β-catenin is associated with poor prognosis of PTC patients and thus contributes to tumor growth. DKK1-secreted protein relocates abnormal expression of β-catenin through Wnt/β-catenin signal transduction, reducing PTC cell survival [55, 56]. The dysregulation of DKK1 gene is a favorable condition for cancer cells to survive and invade. Abnormal expression of DKK1 gene has been detected in a variety of cancer models. In NSCLC, upregulation of DKK1 contributes to cancer, possibly through antagonistic Wnt signaling pathway mediating tumor inhibition of p53 [57]. One study showed that DKK1 was overexpressed in patients with lung and esophageal cancer, leading to poor prognosis in these patients and also becoming a new target for immunotherapy [58]. However, DKK1 is under-expressed in gastric cancer and colorectal cancer, in which DKK1 is regulated by miR-493 and epigenetic silencing, respectively [59]. The activity and expression of DKK1 vary in different cancers, so further exploration of its mechanism is required to verify the prognostic function of DKK1, which can serve as a potential biomarker to accurately predict poor prognosis in patients with these diseases [60].

LDHA is an important energy-metabolizing enzyme with elevated expression in most cancer cells compared to normal tissues [61]. Previous evidence suggests that LDHA mediate tumor spread, invasion, and progression and may be a promising therapeutic target [62–65]. Abnormal expression and upregulation of LDHA are closely associated with a variety of cancers and can be used as a sensitive prognostic factor for lung, liver and pancreatic cancers [66–68]. For example, in gastric cancer (GC), circ-Donson binds to Mir-149-5p, while Mir-149-5p targets LDHA in GC. Down-regulation of circ-Donson inhibits invasion, migration and angiogenesis of tumor cells. However, the high expression of LDHA eventually increased circ-Donson and reduced the inhibition of GC progression [69]. In addition, studies have shown that inhibition of LDHA expression can significantly inhibit cell proliferation, colony formation and migration in lung cancer patients, and enhance their sensitivity to conventional chemotherapy and radiotherapy [70]. Therefore, LDHA is expected to be a promising prognostic indicator for lung cancer treatment. In our study, LDHA was upregulated in patients with a high score, showing a significant association with poor outcomes in LUAD patients.

HMMR, also known as RHAMM/CD168, has a relatively non-negligible role in neurodevelopment, tissue homeostasis and cancer progression [71, 72]. HMMR expression increases in many cancer types and is an important potential prognostic factor in cancers such as Glioblastoma, breast cancer, hepatocellular carcinoma (HCC) [73–75]. HMMR is highly expressed and promotes the growth of LUAD cells. Highly expressed miR-34a-5p induces LUAD cell apoptosis and inhibits cancer cell proliferation by targeting HMMR. However, HCG18 sponges Mir-34A-5p in LUAD to regulate HMMR expression, leading to the rapid development of lung adenocarcinoma and reduced clinical survival time in patients with lung cancer [76]. Previous studies have shown that MPPO-AS1 negatively regulates has-let-7b-5p in lung tumor cells, leading to an over-expression of HMMR and promoting the progression of lung adenocarcinoma [77]. HMMR expression was up-regulated in LUAD, and its high expression was correlated with tumor size and lymph node metastasis. In addition, it was significantly associated with adverse clinical features and prognosis [78], while HMMR knockdown significantly inhibited the invasion ability of LUAD tumor cells. Our results also showed that the expression level of HMMR was closely related to the clinical outcome. The higher HMMR expression, the poorer the OS and clinical prognosis of patients are, making it an important biological marker for the treatment of LUAD.

ANGPTL4, a member of the ANGPTL (ANGPTL1-8) family, is highly expressed in the human vascular system, adipose tissue and intestinal tract, and is involved in the regulation of vascular permeability, angiogenesis and tumorigenesis. In contrast, ANGPTL4 is more important in tumor energy metabolism, antioxidant and metastasis [79]. Studies have shown that ANGPTL4 might have anti-angiogenic and anti-metastatic effects on gastric cancer through the down-regulation of ERK and epigenetic inhibition [80]. However, colorectal cancer (CRC) studies have identified opposite roles of ANGPTL4. DNA methylation-mediated silencing of ANGPTL4 induces the activation of cancer-associated fibroblasts (CAFs) and help CRC transfer through the ERK pathway, enhancing its invasive ability [81]. Moreover, ANGPTL4 can participate in tumor energy metabolism in different NSCLC cells and affect cell proliferation through this process [82]. High expression of ANGPTL4 predicts adverse clinical outcomes in tumors, such as renal clear cell carcinoma, cholangiocarcinoma, melanoma, bladder cancer, and oral cancer [83–87]. In this study, ANGPTL4 was a high-risk gene that increased with tumor progression, suggesting a reduced survival rate and poor prognosis in LUAD patients.

## Conclusion

This study constructed and validated a new prognostic model for LUAD patients based on immune-glycolysis-related genes. The model incorporates clinical prognostic features to predict overall survival in patients diagnosed with LUAD. These findings provide a new method or approach for predicting the prognosis and developing therapeutic strategies for patients with lung adenocarcinoma.

## Supplementary Information

Below is the link to the electronic supplementary material.

Supplementary file1 (DOCX 198 KB)

Supplementary file2 393 genes were selected from survival and expression data of TCGA as prognostic genes (XLS 65 KB)

Supplementary file3 91 DEGs were screened from 443 genes in the TCGA risk group (XLS 31 KB)

## Data Availability

The original contributions presented in the study are included in the article/Supplementary Material. Further inquiries can be directed to the corresponding author.
