# Supplementary material for: Guidelines on lung adenocarcinoma prognosis based on immuno-glycolysis-related genes
Source: Clin Transl Oncol. 2022 Nov 29;25(4):959–75. doi: 10.1007/s12094-022-03000-9 (PMC10025218; doi:10.1007/s12094-022-03000-9)

**Supplementary Fig. S1. TCGA cohort tumor microenvironment score.** (A-B) Immune and stromal scores for both risk groups. (C-D) Relationship between tumor purity and ESTIMATE score in different risk groups.


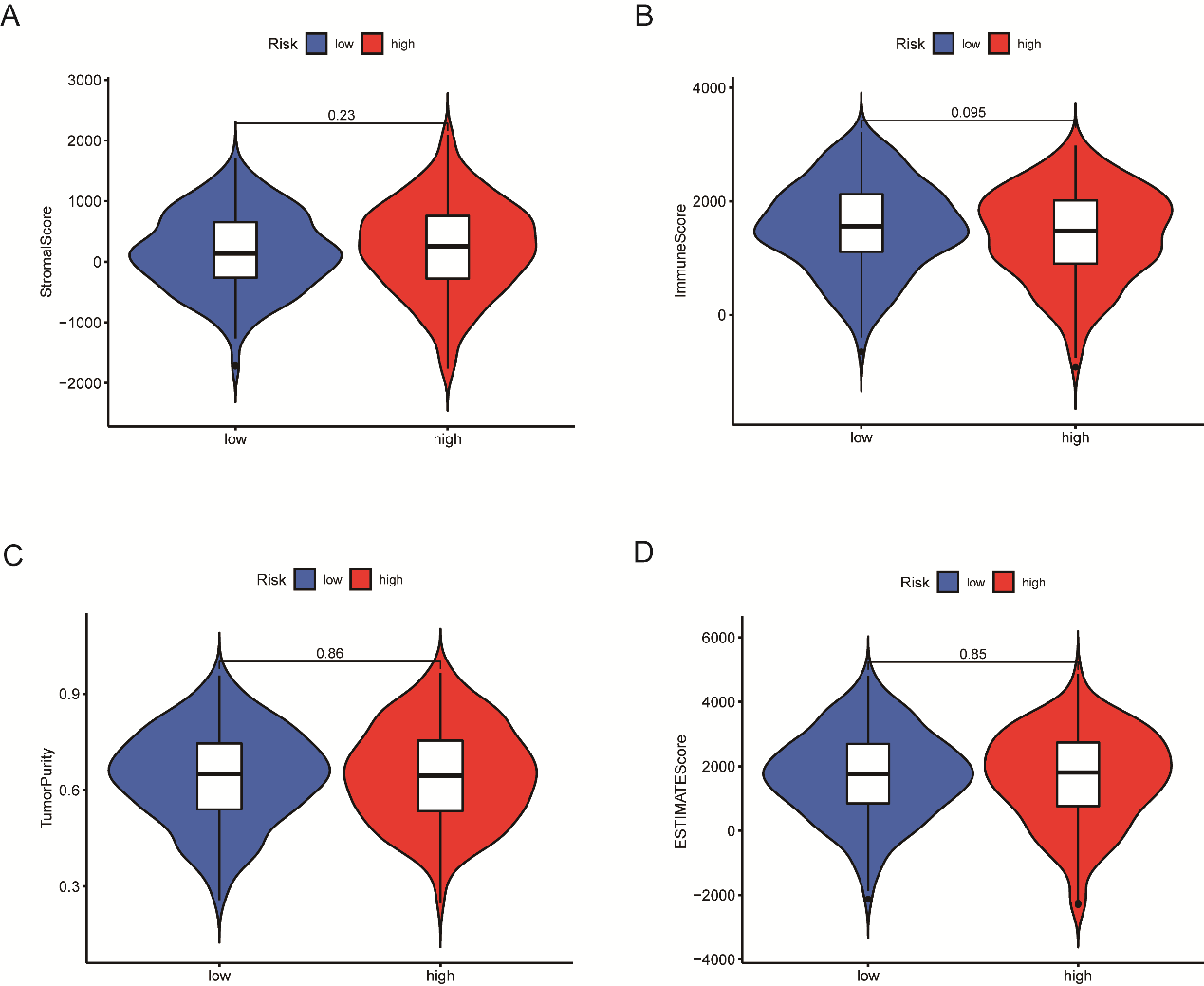


**Supplementary Fig. S2. Prediction of survival by immune characteristics and mutation burden in the TCGA cohort.** (A) Tumor microsatellite instability in the two groups. (B-C) Relationship between IPS of two groups and risk characteristics. (D) Survival curves for the two mutant types in the training group.


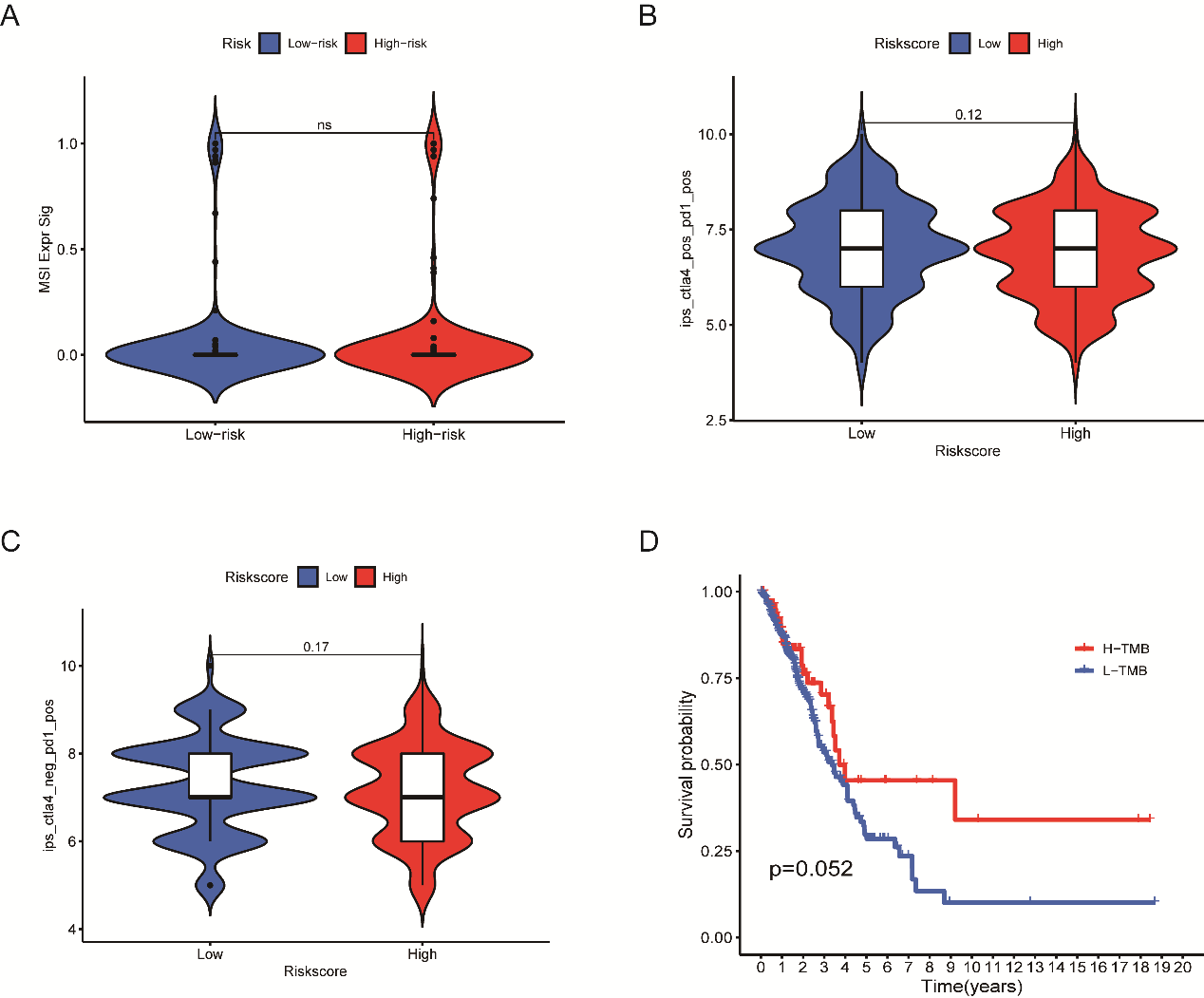

Supplement: Supplementary file 1 — Supplementary file1 (DOCX 198 KB) [file 12094_2022_3000_MOESM1_ESM.docx]
